# Supplementary material for: Creating a Japanese version of the attitudes toward dreams scale: Attitude toward dreams may predict sleep disorders
Source: PLoS One. 2025 Jul 9;20(7):e0326732. doi: 10.1371/journal.pone.0326732 (PMC12240374; doi:10.1371/journal.pone.0326732)
Supplement: S2 File — This file also includes information about copyright and modification conditions. (DOCX) [file pone.0326732.s002.docx]

**A Japanese version of the attitude toward dreams scale**

(2024)

Contact: Shinya Okuyama

Sleep Research Institute

Edogawa University, Chiba Japan

Nagareyama, Komagi 270-0198

okuyamas@edogawa-u.ac.jp

**A Japanese version of the attitude toward dreams scale**

**Permission and Copyright**

This “Japanese version of the attitude toward dreams scale” was created based on a factor analysis of participants' responses to a questionnaire in Japanese. The original version of the scale is in Japanese. Therefore, the questionnaire provided below is an English translation of the original content.

It is considered necessary to examine the validity and reliability of the English version of this scale in a separate study conducted in English-speaking regions.

In this study, we calculated the average of the scores for each factor, and used these mean scores in the analysis.

Although a Japanese version of the attitude toward dreams scale survey is copyrighted, it is available without charge and no written permission is required. This assumes agreement with the following as a consequence of using a Japanese version of the attitude toward dreams scale survey:

● Please refer to the survey using its complete name – a Japanese version of the attitude toward dreams scale and provide the appropriate citation.

● Modifications may be made without our written permission. However, please clearly identify any modifications in any publications that have been made by the users. If you modify the survey, please let us know for our records.

● If you translate the Japanese version of the attitude toward dreams scale into another language, please send us a copy for our records.

**The original questionnaire of a Japanese version of the attitude toward dreams scale**

|  | **各項目の内容が，あなた自身にどの程度あてはまるかについて回答してください．** | | | | | | | |
| --- | --- | --- | --- | --- | --- | --- | --- | --- |
|  |  | **全くあて はまらない** |  |  |  |  |  | **非常によく あてはまる** |
| **因子 1;  夢の意味** | **1. 夢で自分を知ることが出来ると思う．** | 1 | 2 | 3 | 4 | 5 | 6 | 7 |
|  | **2. 夢を理解することより、その人の生活をよりよくすることができると思う．** | 1 | 2 | 3 | 4 | 5 | 6 | 7 |
|  | **3. 自分の見た夢をもっとよく理解したい．** | 1 | 2 | 3 | 4 | 5 | 6 | 7 |
|  | **4. 夢はとても大切なものだと思う．** | 1 | 2 | 3 | 4 | 5 | 6 | 7 |
|  | **5. 夢はその人の性格をあらわすと思う．** | 1 | 2 | 3 | 4 | 5 | 6 | 7 |
| **因子 2; 夢の無意味** | **1. 自分の夢を真剣に受け止めていない．** | 1 | 2 | 3 | 4 | 5 | 6 | 7 |
|  | **2. 夢を見たとしても、それが役に立つことはない．** | 1 | 2 | 3 | 4 | 5 | 6 | 7 |
|  | **3. 夢には意味がないと思う．** | 1 | 2 | 3 | 4 | 5 | 6 | 7 |
|  | **4. 夢は脳で作られた無意味な産物である．** | 1 | 2 | 3 | 4 | 5 | 6 | 7 |
|  | **5. 夢を分析する必要はない．** | 1 | 2 | 3 | 4 | 5 | 6 | 7 |

**English translation: Questionnaire based on the Japanese version of the attitude toward dreams scale**

|  |  | **Please indicate to what extent this applies to you.** | | | | | | |
| --- | --- | --- | --- | --- | --- | --- | --- | --- |
|  |  | **Not at all  applicable** |  |  |  |  |  | **Very  applicable** |
| Factor 1; Meaning of Dreams | 1. I think that one can learn about oneself through dreams. | 1 | 2 | 3 | 4 | 5 | 6 | 7 |
|  | 2. I think that one can improve one's life by understanding their dreams. | 1 | 2 | 3 | 4 | 5 | 6 | 7 |
|  | 3. I want to better understand the dreams I have had. | 1 | 2 | 3 | 4 | 5 | 6 | 7 |
|  | 4. I think that dreams are very important. | 1 | 2 | 3 | 4 | 5 | 6 | 7 |
|  | 5. I think that dreams reveal the dreamer's personality. | 1 | 2 | 3 | 4 | 5 | 6 | 7 |
| Factor 2; No Meaning of Dreams | 1. I do not take my dreams seriously. | 1 | 2 | 3 | 4 | 5 | 6 | 7 |
|  | 2. Even if I have dreams, they cannot be helpful. | 1 | 2 | 3 | 4 | 5 | 6 | 7 |
|  | 3. I think that dreams have no meaning. | 1 | 2 | 3 | 4 | 5 | 6 | 7 |
|  | 4. Dreams are a meaningless product of the brain. | 1 | 2 | 3 | 4 | 5 | 6 | 7 |
|  | 5. There is no need to analyze dreams. | 1 | 2 | 3 | 4 | 5 | 6 | 7 |
