# Supplementary material for: Creating a Japanese version of the attitudes toward dreams scale: Attitude toward dreams may predict sleep disorders
Source: PLoS One. 2025 Jul 9;20(7):e0326732. doi: 10.1371/journal.pone.0326732 (PMC12240374; doi:10.1371/journal.pone.0326732)
Supplement: Attachment — Submitted filename: Review.docx [file pone.0326732.s003.docx]

**Creating a Japanese version of the attitude toward dreams scale: Attitude toward dreams may predict sleep disorders**

**General Comment**
As a general remark, the study is interesting, and the significant effort in adapting scales related to dreams and sleep disorders to Japanese culture is commendable. The authors should be congratulated for this. However, the manuscript has some methodological issues and problems presenting results, described below in the affected sections.

**Title**

**The title is attractive but it certainly does not reflect the results obtained. I suggest modifying the second part of the title according to my comments under the "Conclusion" section.**

**Introduction**
This section is excessively brief and includes information that should be placed in other sections. Only a few studies by Schredl and Kodama on attitudes toward dreams are mentioned, but there is no mention of these scales' utility or practical applications. Furthermore, no references are made to studies that relate these scales to sleep disorders.

The study employs many other scales and instruments (lines 84-92), but none are mentioned in the introduction.

Therefore, the authors must include references to previous studies that have linked the attitude scales with the instruments used in the study (e.g., RBD, STOP-Bang, Restless Legs Syndrome, Athens Insomnia Scale, PSQI-J, SF-8, NEO-FFI, Metamemory in Adulthood Questionnaire, Dream Propensity Scale [22]; Munich Chronotype Questionnaire for biological rhythm assessment, Nightmare Distress Questionnaire).
This would help justify the relationship between such variables in the study and provide background on possible correlations with the other scales.

At the end of the Introduction, only the aim of validating the Japanese attitude scale is mentioned. The authors should provide, as an Appendix, the items or questions and the response options of the Japanese scale they intend to validate, along with their English translation. Furthermore, the study suggests at least one other objective: to verify whether this scale can help to predict sleep disorders.

The authors need to explain all the objectives of the study clearly. They should also clarify why they believe that attitudes toward dreams may predict sleep disorders, i.e., what specific hypothesis they are testing. They need to state whether attitudes can predict any sleep disorder or only specific ones.

Moreover, lines 57-58 present data on the study participants, which should not be included in the introduction. Similarly, lines 58-62 contain content that should be moved to the **Method** section.

**Participants**
In this section, it would be useful to report the mean age and standard deviation for each gender in addition, to the age range of the participants (20-70 years).

Line 71 suggests that not all survey responses were used, as only those considered consistent were selected. The authors should specify the entire number of excluded responses and the precise selection criteria employed.

**Survey Items**
Reference [17] corresponds to a scale developed in Greek and translated into English. You have used it in your study, but it is not described whether this scale has been validated for Japanese. You need to explain how this issue was addressed.

**Attitudes Toward Dreams**

Several issues:

- **Reference [9]**
  The Mannheim Dream Questionnaire attitude scale was created in German and subsequently translated and validated in many studies, however, it is not clear into which language it was translated for the Japanese context or whether any validation was performed on that translation. Additionally, the original scales had five response options (ranging from "Not at all" to "Totally"), whereas you state that you used six. Therefore, it is not understood, nor have you justified, how you validated the results of this scale. You should justify and explain which response option was added.
- **Reference [2]**
  This reference pertains to a study on gender differences in the frequency of dream recall, but it does not validate any specific scale related to attitudes toward dreams. You should explain more clearly which items of this scale you included, how they relate to attitudes, who the original authors are, and how it was translated and validated into Japanese.
- **Reference [4]**
  The attitude scale you use in this study is in English, and you do not explain how it was translated and validated into Japanese.
- **Reference [11]**
  This is a study in Japanese, which corresponds to a key scale for your study. It would be very useful if you could provide an English version of this scale as an appendix to evaluate the differences compared to the other scales used.

**Analysis**
The fit indices you use are appropriate, but you do not include others that are methodologically considered very suitable for evaluating model fit in CFA, such as CFI, TLI, and SRMR (See: Schumacker, R. E., & Lomax, R. G. (2016). A Beginner’s Guide to Structural Equation Modeling. Routledge).

In lines 113 to 115, you describe the formula for the AIC index, but the other indexes. This information is not particularly relevant. If you are going to include formulas, you should do so for all the indexes employed or purely omit the formulas since it is unnecessary if references are included.

An issue is not addressed, and it should be clarified. It appears that one of the attitude scales relevant to Japanese culture is the one by Kodama (Ref. [11]), lines 51 to 53, which identified three factors regarding attitudes toward dreams among Japanese participants: Factor 1 (meaning and utilization of dreams), Factor 2 (value of dreams), and Factor 3 (influence of dreams).

Why was this hypothesis not tested for the CFA in the proposed scale? Or, if it was, the results should be provided.

**Results**
The first section presents Table 1, which compares the DRF with a study by Schredl (2020). However, the necessity or usefulness of these calculations and comparisons for the aims of this study is unclear. You should either justify the need for this table or consider omitting it.

Table 2 presents the standardized factor loadings for the items on the attitudes toward dreams scale. However, you need to adequately explain which attitude scale you are referring to. Specifically, you should clarify whether the 10-item attitudes toward dreams scale is one you have created or if it corresponds to a scale from previous studies.

Table 3 shows correlations between factor scores of the Attitudes-Toward-Dreams Scale and other scales. First, you should specify which statistical correlation coefficient was used (e.g., Pearson, Spearman?). Second, it seems that the reader should interpret that a single measure has been derived for each factor. If this is the case, it should be explicitly stated and the method of calculation clarified—whether it was done by calculating the arithmetic mean of the items or using another formula.

Figures 2, 3, and 4 present a lot of information related to age and gender, but no initial hypothesis has been made regarding these variables. Including so many figures concerning a secondary aspect of the study creates confusion for the reader. If you intend to perform an invariance analysis of the results, it would be better to test whether the CFA results replicate for each gender and age group separately. Otherwise, you should simplify these results and provide only one figure.

**Discussion**
In lines 229 to 231, you state that Factor 1 reflects an attitude of seeking “hidden” meanings in dreams. However, this is the first mention of "hidden" or "interpretation" in your manuscript. You need to explain why you have reached this conclusion, as it is not directly related to the contents of the scale.

In line 233, you analyze the correlations between each factor and certain variables related to sleep disorders. First, it is unclear why you include the correlation with the DRF here, as it is not considered a disorder. Second, you state that "Our results suggest that attempting to find meaning in dreams may be associated with unhealthy sleep patterns," but "unhealthy sleep patterns" (which refer to habits) are not the same as "sleep disorders" (which are medical conditions). This confusion may have been due to language differences, but you should pay attention to the distinction between meanings in English.

In any case, the highest correlations found are very low associations (less than .30), and it is not appropriate to infer causality or suggest that attitudes can predict sleep disorders. Maybe you can reconsider the manuscript title using something like: “Attitudes towards dreams can be associated with some sleep disorders”.

It would be interesting to expand this section and compare the results of this study with those from other studies on attitudes toward the two factors in previously referenced studies. For example, compare your results with those of Kodama [11] or with the various studies by Schredl over different years and countries.

**Conclusion**
You state that "the results also suggest that attitudes toward finding meaning in dreams may be associated with poorer sleep quality and lower quality of life." However, this is the first mention of "quality of life" in the manuscript. It is not advisable to generalize this concept at the end of the manuscript, as it has not been discussed earlier. A more precise and scientific approach should focus solely on the results obtained from the new attitude scale toward dreams, validated in Japanese and for Japanese culture.

Based on my understanding, and considering the limited associations observed with variables related to sleep disorders, it would be more appropriate to present this as an exploratory study, with results that could serve as a foundation for future research in this field, rather than a confirmatory study.
