# Supplementary material for: Creating a Japanese version of the attitudes toward dreams scale: Attitude toward dreams may predict sleep disorders
Source: PLoS One. 2025 Jul 9;20(7):e0326732. doi: 10.1371/journal.pone.0326732 (PMC12240374; doi:10.1371/journal.pone.0326732)
Supplement: Attachment — Submitted filename: Review PONE.docx [file pone.0326732.s004.docx]

Review PONE-D-24-51943

**Overall comment:**

It is an interesting paper, where a scale about people’s attitudes toward dreams has been developed and tested in a Japanese population. It is of interest and relevance for sleep medicine but could do with some clarifications to make it easier for the reader to follow.

**Abstract:**

The aim of the study is described differently in the abstract compared to in the paper. Also, in the aim described in the abstract, the authors write about relevant factors influencing attitudes toward dreams. What does relevant factors mean in this context? It would be clearer if the authors specified the factors.

**Introduction:**

Page 3, line 54-56: here the aim is presented once again, adding a possible development of a multidimensional scale (which the one in the abstract does not say). Since scale development seems to have been the general idea from the start, this should be added to the aim in the abstract and I also think that ‘if necessary’ (page 3, line 55) can be omitted.

Page 3, line 57-62:

Consider moving this text to the Materials and methods section instead. It seems more to describe what the authors have done rather than provide a rationale for the study.

**Materials and methods**

*Participants and data collection*

Recruitment and data collection happened in only 5 days, did participants provide their written, informed consent in this time as well, or was consent collected before sending out the survey?

Did the authors consider any reminders?

How were the participants selected, was the sample stratified in some way?

*Attitudes toward dreams*

Page 5

I’d like a more thorough description of the scale development. How did the author decide on what items to pick from each of the previous questionnaires?

What was the motive for changing the response-alternatives from the original scale?

*Analysis*

Page 5

As far as I can tell, the analysis seems to be alright, but perhaps the authors would consider starting with describing what analyses they will use and stepwise describe the scale development. This could be a way to more clearly describe how they chose items from the other questionnaires to form a new one.

**Results**

It would have been nice with a table presenting the participants of the study, describing the distribution between sex, mean age, geographical area, mean score of the scales used.

Page 6, Table 1

This table shows a comparison between the current study sample and a previous study by Schredl, however, it seems that there were different number of response-alternatives in the studies, or why is it a blank in the column describing the data of Schredl? How should that be interpreted, that is, with fewer options, how would they have responded compared to the present study? I don’t think you can compare straight across the samples given these circumstances.

Page 7, Table 2

Just to be clear, table 2 shows the scale that was developed in the current study. Maybe this could be clearly stated in the text.

Page 8, line 153

Just a minor comment, the authors should not present Table 4 in the text before presenting Table 3 in the text.

Page 9, line 167-169

There are several measures mentioned in the text below the table, but where can I see the data from those measures (e.g. blood pressure, BMI, age, neck circumference, gender). Maybe this could be added to a table describing the participants of the study?

Several of the correlations presented in table 3 were statistically significant, however the correlations were weak in general, which is important to remember when considering the findings.

Page 10, Table 4

Could the authors please add the maximum score for each item, just so that the reader can evaluate what the mean-values indicate.

Page 11, line 212

The text says there is a difference between sex for dream frequency, but shouldn’t it be dream-recall frequency? I believe it is only subjective data collected, i.e. what the persons remember regarding their dreams, no EEG that can tell objectively if and how much they have dreamt.

The term dream frequency occurs again in page13, line 260, 261 and 262.

Discussion

Page 12, line 219

Here is a third version of the aim, stating that the purpose of the study was to develop a scale. Maybe the authors could agree on one of the three versions of the aim and then use that throughout the paper for clarity.

Page 12, line 235

Here the authors discuss the correlations between the Attitudes toward dreams scale and other scales, pointing out that there are statistically significant correlations, but it should be discussed that they are weak and what that implies in terms of interpreting the clinical significance of the correlations.

Page 13, line 256-257

It is suggested that having a high interest in dreams in older age may indicate susceptibility to RBD. How so? In RBD vivid, frightful dreams may occur, but not always remembered by the dreamer. Another feature is that the normal REM-sleep muscle atony is disconnected, and the person may ‘live’ their dream. I have never heard that remembering one’s dreams or trying to find meaning in them would be related to RBD. The authors may want to add references that supports their statement or change it.

**Limitations of the study**

Page 13, line 267

I do not understand why the survey being online would have caused participants to misunderstand questions, if the authors please would elaborate on that. I think that if a question is misunderstood it is about the wording and that would be the same for both paper questionnaire and online surveys.

Page 14, line 272

The results may have been influenced by cultural factors of Japan. I do not see this as a limitation, as the authors clearly state that they wish to investigate attitudes toward dreams in Japanese men and women. (I understood it as to really consider possible cultural factors, that may be specific for this population). However, it would be interesting if the authors presented some cultural differences found through the study and compare these to previous studies in other poplations.
