# Supplementary material for: Creating a Japanese version of the attitudes toward dreams scale: Attitude toward dreams may predict sleep disorders
Source: PLoS One. 2025 Jul 9;20(7):e0326732. doi: 10.1371/journal.pone.0326732 (PMC12240374; doi:10.1371/journal.pone.0326732)
Supplement: Attachment — Submitted filename: Response_to_Reviewers2025.03.12.docx [file pone.0326732.s006.docx]

「PLOS ONE」 Reviewer’s comments & Response

File Inventory Manuscript Number: PONE-D-24-51943

DOI: Unassigned
Shinya Okuyama (JAPAN): "Creating a Japanese version of the attitude toward dreams scale: Attitude toward dreams may predict sleep disorders"

―――――――――――――――――――――――――――――――――――――

**Reviewer #1:** Overall, the paper is well written and the authors' effort to incorporate and validate Western instruments into Japanese culture is interesting and deserves to be supported because it generally improves scientific progress in general. However, the study has some important methodological problems that need to be corrected or adequately explained. In an attempt to incorporate instruments into Japanese culture, the authors have made an effort that may have been too optimistic and with methods that statistically favor obtaining associations, such as performing a CFA followed by an EFA. The fit indices employed by the authors are not particularly stringent. This leads them to exaggerated conclusions, including the title.

【Response】We thank you for your insightful review of our manuscript and valuable comments. It seems there was a misunderstanding about the order of exploratory factor analysis (EFA) and confirmatory factor analysis (CFA). In this study, we first performed EFA to explore the factor structure based on the data. After that, we conducted CFA to verify whether the explored factor structure fits the data. This approach follows the standard procedure in factor analysis, and by confirming the factor structure explored through EFA with CFA, we can obtain more robust results. Therefore, we believe that we have not used a statistically biased method regarding the order of EFA and CFA. We have added the analysis procedure to the “Methods” section.

―――――――――――――――――――――――――――――――――――――

**Reviewer #2:** Review PONE-D-24-51943

Overall comment:

Thank you for the opportunity to read and review this paper. It is an interesting paper, where a scale about people’s attitudes toward dreams has been developed and tested in a Japanese population. It is of interest and relevance for sleep medicine but could do with some clarifications to make it easier for the reader to follow.

Abstract:

The aim of the study is described differently in the abstract compared to in the paper. Also, in the aim described in the abstract, the authors write about relevant factors influencing attitudes toward dreams. What does relevant factors mean in this context? It would be clearer if the authors specified the factors.

【Response】We thank you for your insightful review of our manuscript and valuable comments. Our point-by-point responses are provided below.

We appreciate your suggestion to clarify the aim of our study and specify the "relevant factors" mentioned in the abstract.

In response to your comment, we have revised the abstract to explicitly specify the factors examined in our study. These include: dream attitudes (Factor 1: meaning of dreams, Factor 2: no meaning of dreams), dream-recall frequency, various sleep-related variables (e.g., Pittsburgh Sleep Quality Index, sleep apnea, restless legs syndrome, insomnia, and rapid eye movement sleep behavior disorder), personality traits, and quality of life. We believe that this revision provides a clearer understanding of the factors studied and aligns the aim of the study more closely with the content of the paper. We hope this revision addresses your concern.

Introduction:

Page 3, Line 54-56: here the aim is presented once again, adding a possible development of a multidimensional scale (which the one in the abstract does not say). Since scale development seems to have been the general idea from the start, this should be added to the aim in the abstract and I also think that ‘if necessary’ can be omitted.

【Response】To ensure consistency between the Abstract and the text regarding the purpose of the study, we should have explicitly stated the multidimensional development of the “Attitude Toward Dreams Scale” in the Abstract as well. Therefore, we have added the sentence “We aimed to develop a dream attitude scale” to the Abstract, and we have deleted the phrase “as needed”. The modification is as follows:

Page 2, Line 17-22: “Therefore, in this study, we aimed to identify the relevant factors influencing attitudes toward dreams among Japanese participants. These factors included dream attitudes, dream-recall frequency, various sleep-related variables (Pittsburgh Sleep Quality Index, sleep apnea, restless legs syndrome, insomnia, and rapid eye movement sleep behavior disorder), personality traits, and quality of life. In addition, we also aimed to develop a new dream attitude scale."

Page 3, Line 56-58: “Therefore, this study aimed to identify the factors specific to Japanese participants and, if necessary, develop a multidimensional scale for measuring “attitudes toward dreams” by creating new questionnaire items based on existing dream attitude scales.”

Page 3, Line 57-62: Consider moving this text to the Materials and methods section instead. It seems more to describe what the authors have done rather than provide a rationale for the study.

【Response】Indeed, the information on dream recurrence frequency, age, sleep habits, personality traits, and metamemory should be explained as part of the research methodology, and it would be more appropriate to move it to the Materials and Methods section as a description of the methodology rather than a description of the purpose of the study. Therefore, this section has been moved to the Materials and Methods section and the relevant section has been revised (Page 3, Lines 61-67).

Materials and methods

Participants and data collection

Recruitment and data collection happened in only 5 days, did participants provide their written, informed consent in this time as well, or was consent collected before sending out the survey?

【Response】Data collection for this study was contracted to a web-based research firm, Cross Marketing, Inc. Because the survey was conducted online, consent was confirmed online rather than in writing. At that time, those who did not consent were not allowed to respond to the survey.

Did the authors consider any reminders?

【Response】The data collection for this study was outsourced to Cross Marketing Inc, a web survey company. We are unaware of whether reminders were sent. However, considering that the data was collected within a 5-day period, it seems unlikely that reminders were issued.

How were the participants selected, was the sample stratified in some way?

【Response】The participants in this study were randomly selected from individuals who had previously participated in another survey conducted by Cross Marketing (a web survey company) within the past year. The sample included men and women in their 20s to 70s, and the survey URL was closed once the target sample size was reached for participants who completed the entire questionnaire. However, to account for potential data loss during the data cleaning process, we collected approximately 10% more data than the intended sample size. In the end, Cross Marketing delivered data from 1,680 valid responses, randomly selected from the pool. The breakdown of the 1,680 participants was specified by us, with 140 men and 140 women in each age group from the 20s to 70s.

Attitudes toward dreams

Page 5

I’d like a more thorough description of the scale development. How did the author decide on what items to pick from each of the previous questionnaires?

【Response】In previous studies, the standard factor structure of the Attitudes Toward Dreaming Scale was considered to be based on “positive and negative attitudes toward dreams,” so the Mannheim Dream Questionnaire (2014), Attitudes toward Dreaming (2019), and Attitudes Toward Dreams Scale (2010) were employed. Furthermore, we wondered if we could create an Edogawa University version of the original Attitudes Toward Dreaming scale by adding and validating Dr. Kodama's Dream Attitudes Scale (2000), which had published a factor structure other than “positive and negative attitudes toward dreams” in this study.

What was the motive for changing the response-alternatives from the original scale?

【Response】In the attitude questionnaire toward dreams conducted in this study, a 7-point scale was used. Schredl used a 5-point scale and Kodama used a 4-point scale, so it was necessary to unify them in this study. Weng (2004) reported that "whether language labels are provided for all categories or only for the endpoints, having fewer response categories results in lower reliability." Additionally, Krosnick & Presser (2010) provided comprehensive guidelines for survey questionnaire and scale design, stating that a 7-point scale "allows respondents' opinions to be more accurately reflected by including a middle option." However, neither of these studies concluded that the 7-point scale is superior to other methods. Nevertheless, in this study, in order to enhance the reliability of the survey results, the number of response categories was increased.

Additionally, the following two sentences were added under “Attitudes toward dreams” in the Materials and Methods section.

Page 5, Lines 106-108: “Since these studies used the five-point scale [2, 4, 9] or the four-point scale [11], the questionnaire in this study was standardized to a seven-point scale.”

　Page 5, lines 109-115: “Weng [25] reported that "whether language labels are provided for all categories or only for the endpoints, having fewer response categories results in lower reliability." Additionally, Krosnick and Presser [26] provided comprehensive guidelines for survey and scale design, noting that a seven-point scale "can more accurately reflect respondents' opinions by including a middle option." However, neither of these studies concluded that the seven-point scale is superior to other methods. Nevertheless, in this study, a seven-point scale was used in the survey on attitudes toward dreams to enhance the reliability of the results.”

Analysis

Page 5

As far as I can tell, the analysis seems to be alright, but perhaps the authors would consider starting with describing what analyses they will use and stepwise describe the scale development. This could be a way to more clearly describe how they chose items from the other questionnaires to form a new one.

【Response】In this study, we developed a new “Attitude Toward Dreams scale" based on four previous studies. The flow of analysis on the results of the questionnaire regarding attitudes toward dreams has been added to the main text.

Pages 5-6, Lines 118-129: “The results of the questionnaire on the Attitude Toward Dreams scale were first subjected to exploratory factor analysis (EFA) to extract the factor structure. Parallel analysis and eigenvalue criteria were used to determine the number of factors, retaining items with factor loadings of 0.4 or higher. As a result, several factors were clearly extracted, and the scale structure was conceptually clarified. Based on the EFA findings, confirmatory factor analysis (CFA) was conducted to assess the goodness of fit of the scale. In the CFA, model fit indices (the goodness of fit was assessed using the goodness-of-fit index, root mean square error of approximation, chi-square minimum fit index (CMIN), and Akaike’s information criterion (AIC)) were used to verify how well the structure fit the data. Items with factor loadings of 0.5 or higher were selected, confirming that the scale appropriately measured the intended constructs. For the evaluation of reliability and validity, Cronbach's alpha was used for each factor. Additionally, Bonferroni correction was applied for multiple comparisons to confirm statistical significance.”

Results

It would have been nice with a table presenting the participants of the study, describing the distribution between sex, mean age, geographical area, mean score of the scales used.

【Response】I have created Table 1, titled “Number of participants in each area of Japan.”

The accompanying description for Table 1 has also been revised:

Pages 6-7, Lines 145-148: “Table 1 shows a distribution of participants recruited into the study from eight areas of Japan. Japan has 47 prefectures, which are summarized into eight areas. The population distribution in this study is similar to the actual distribution. There was no prominent skewing in terms of sex or mean ages among these areas.”

Page 6, Table 1

This table shows a comparison between the current study sample and a previous study by Schredl, however, it seems that there were different number of response-alternatives in the studies, or why is it a blank in the column describing the data of Schredl? How should that be interpreted, that is, with fewer options, how would they have responded compared to the present study? I don’t think you can compare straight across the samples given these circumstances.

【Response】As stated in the Methods section, this study used a 7-point scale survey. Therefore, as you pointed out, it was not appropriate to compare the results of this study with those of Schredl. However, we wanted to show the difference between the two sides, as their results were very different. I have added a note in the text clarifying that the inclusion of Schredl’s results in the table was solely for reference.

I have added the following explanation regarding Table 2.

Page 7, Lines 153-156: “Table 2 shows the distribution of dream-recall frequency for the total sample and based on sex. Since the number of choices in the surveys used in this study and Schredl’s study [5] is different, they cannot be compared. Therefore, the results of both studies are shown in the same table for reference only.”

Page 7, Table 2

Just to be clear, table 2 shows the scale that was developed in the current study. Maybe this could be clearly stated in the text.

【Response】The text describing Table 3 “presents the newly developed Attitude to Dreams scale developed in this study and the standardized factor loadings of the items on the scale.” The text has been modified as follows.

Page 8, Lines 150-161: “Table 3 shows the newly developed Attitude Toward Dreams scale in this study and the standardized factor loadings for each scale item.”

Page 8, line 153

Just a minor comment, the authors should not present Table 4 in the text before presenting Table 3 in the text.

【Response】Table 1 is newly added, and thus, the previous Table 3 has been changed to Table 4 and Table 4 to Table 5. I fixed the order in which Tables are presented.

In addition, an explanation of the results shown in Table 5 is noted below.

Page 11, Lines 202-205: “Table 5 shows the mean scores and standard deviations for each element and Attitude Toward Dreams scale item, with a maximum value of 7. The mean score for Factor 1 was 3.59 ± 1.30, while that for Factor 2 was 4.29 ± 1.23. The mean score for Factor 1 was significantly lower than that for Factor 2 (t (1477) = 16.007, p < 0.001).”

Page 9, line 167-169

There are several measures mentioned in the text below the table, but where can I see the data from those measures (e.g. blood pressure, BMI, age, neck circumference, gender). Maybe this could be added to a table describing the participants of the study?

【Response】We did not investigate “high blood pressure,” “body mass index,” or “neck circumference” as you pointed out. It was our mistake to describe this. I am very sorry about that. Information on age and gender differences is provided in Table 1.Several of the correlations presented in table 3 were statistically significant, however the correlations were weak in general, which is important to remember when considering the findings.

【Response】We are aware of the overall low correlation in the data of this study as per your comment. We indicate this in the text.

Page 10, Table 4

Could the authors please add the maximum score for each item, just so that the reader can evaluate what the mean-values indicate.

【Response】I have indicated a maximum score of “7” in the legend of the new Table 5 (Page 12, Line 209).

Page 11, line 212

The text says there is a difference between sex for dream frequency, but shouldn’t it be dream-recall frequency? I believe it is only subjective data collected, i.e. what the persons remember regarding their dreams, no EEG that can tell objectively if and how much they have dreamt.

The term dream frequency occurs again in page13, line 260, 261 and 262.

【Response】As you mentioned, I believe “dream-recall frequency” is the more appropriate expression. I have revised all instances of “dream frequency” in the text to “dream-recall frequency.”

Discussion

Page 12, line 219

Here is a third version of the aim, stating that the purpose of the study was to develop a scale. Maybe the authors could agree on one of the three versions of the aim and then use that throughout the paper for clarity.

【Response】We believe that the main purpose of this study was only to develop a new scale structure.

Page 12, line 235

Here the authors discuss the correlations between the Attitudes toward dreams scale and other scales, pointing out that there are statistically significant correlations, but it should be discussed that they are weak and what that implies in terms of interpreting the clinical significance of the correlations.

【Response】The results relating attitudes toward dreams to various variables in this study were weakly correlated, as you noted. This also indicates that our claims may be limited, and we have added a limitation to that effect.

Page 13, line 256-257

It is suggested that having a high interest in dreams in older age may indicate susceptibility to RBD. How so? In RBD vivid, frightful dreams may occur, but not always remembered by the dreamer. Another feature is that the normal REM-sleep muscle atony is disconnected, and the person may ‘live’ their dream. I have never heard that remembering one’s dreams or trying to find meaning in them would be related to RBD. The authors may want to add references that supports their statement or change it.

【Response】In this study, we reported that a high interest in dreams in older age may indicate susceptibility to REM sleep behavior disorder (RBD). However, we do not yet fully understand the underlying mechanism. Previous studies have not reported a link between the tendency to find meaning in dreams and RBD. Additionally, considering the results of our study (which showed weak correlations between high interest in dreams and several scales, including RBD), we believe the implications are limited. Further detailed investigation is needed to better understand this mechanism. The above points were added as a limitation. The text added to the Limitation section is as follows.

Page 15, Lines 292-296: “In this study, since the correlation noted between Factor 1 and RBD was weak, its implications may be limited. Furthermore, previous studies have not reported a link between the tendency to find meaning in dreams and RBD, and the underlying mechanism for such a relationship remains unclear. Therefore, we believe that further detailed investigation in this regard is needed.”

Limitations of the study

Page 13, line 267

I do not understand why the survey being online would have caused participants to misunderstand questions, if the authors please would elaborate on that. I think that if a question is misunderstood it is about the wording and that would be the same for both paper questionnaire and online surveys.

【Response】You are correct that misunderstandings can occur in both online and paper surveys due to wording issues. However, we included this limitation specifically because online surveys may have unique factors that could influence participants' understanding, such as the lack of an interviewer to clarify questions in real time. Additionally, participants may be distracted or may not pay close attention while completing an online survey, leading to potential misinterpretations of questions. In contrast, in paper surveys, participants might have more time and the opportunity to seek clarification if needed. However, we agree that the way questions are worded is the main factor influencing misunderstandings, regardless of whether the survey is conducted online or on paper. We appreciate your valuable insight.

Page 14, line 272

The results may have been influenced by cultural factors of Japan. I do not see this as a limitation, as the authors clearly state that they wish to investigate attitudes toward dreams in Japanese men and women. (I understood it as to really consider possible cultural factors, that may be specific for this population). However, it would be interesting if the authors presented some cultural differences found through the study and compare these to previous studies in other populations.

【Response】We appreciate your suggestion that the purpose of this study was to investigate the attitudes toward dreams among Japanese participants. We considered the possibility that the results may not be generalizable to groups with different cultural backgrounds and that cultural factors might have influenced the observed attitudes. However, it is also true that we are intrigued by the fact that the results closely resemble those obtained in Western studies. We agree with your suggestion to further explore cultural differences, and comparing our findings with those from other groups would be interesting. In future research, it would be valuable to investigate whether the same attitudes toward dreams can be applied to people from different cultural backgrounds. Furthermore, the section of the text you pointed out has been removed.
