# Supplementary material for: Creating a Japanese version of the attitudes toward dreams scale: Attitude toward dreams may predict sleep disorders
Source: PLoS One. 2025 Jul 9;20(7):e0326732. doi: 10.1371/journal.pone.0326732 (PMC12240374; doi:10.1371/journal.pone.0326732)
Supplement: Attachment — Submitted filename: Revised Response to Reviewers2025.05.10.docx [file pone.0326732.s007.docx]

PONE-D-24-51943R1

Creating a Japanese version of the Attitudes Toward Dreams scale: Attitude toward dreams may predict sleep disorders

[Responses to Reviewers' comments/questions]

１．Reviewer #1: I noticed that you have included the analysis procedure at the end of the 'Methods' section. While this is adequate, for future submissions, I would recommend adding a dedicated section that provides a detailed explanation of the statistical technique employed. Additionally, it may be beneficial to suggest the use of distinct data samples for the Exploratory Factor Analysis (EFA) and Confirmatory Factor Analysis (CFA)

**Response:** Thank you for your suggestion to include a separate section with a more detailed explanation of the statistical methods used. To address your comments, we added a statement to the limitations section clarifying that both the exploratory factor analysis (EFA) and confirmatory factor analysis (CFA) were conducted using the same dataset. We will adopt your recommended approach for data analysis and reporting in future, more advanced versions of this work. The following text has been added to the limitations section (page 19, lines 361–365).

“*Additionally, both the EFA and CFA were conducted using the same dataset. While this approach was deemed appropriate for the initial development of the scale, further validation is necessary. Future studies should aim to replicate the identified factor structure using independent samples to establish the generalizability and stability of the scale across diverse populations*.”

Reviewer #3: The article is interesting and seems to have improved since the first version. However, there is still a need for improvement. Moreover, I noted that the authors did not answer the reviewers' point-by-point:

1) It is necessary to specify the aim of the study, because the authors still report three different versions in the text.

Page 2, lines 17-18 " we aimed to identify the relevant factors influencing attitudes toward dreams among Japanese participants";

Page 3, lines 56-58, "this study aimed to identify the factors specific to Japanese participants and, if necessary, develop a multidimensional scale for measuring “attitudes toward dreams” by creating new questionnaire items based on existing dream attitude scales”;

Page 13, line 246, "the purpose of this study was to develop a Japanese version of the attitudes-toward-dreams scale."

**Response:** Thank you for pointing out the inconsistencies in the description of the study’s aim. To address this issue and improve clarity, we revised the text to provide a unified and consistent statement of the aim of the study throughout the manuscript. The statements have been revised as follows.

・Page 2, lines 17–19: “*This study aimed to develop a Japanese version of a scale to assess attitudes toward dreams and to examine factors related to Japanese participants' attitudes toward dreams*.”

・Page 3, line 55–57: “*Therefore, this study aimed to develop a Japanese version of a scale to assess attitudes toward dreams and to examine the factors associated with Japanese participants' attitudes toward dreams*.”

・Page 16, lines 303–304: “*This study aimed to develop a Japanese version of the Attitude Toward-Dreams scale and to examine the factors associated with Japanese participants’ attitudes toward dreams.*”

2) It is not clear how you selected the final sample. How did you get from 1680 to 1478 participants? What do you mean in line 74 “who provided consistent responses about sleep-related factors?" Please explain the inclusion and exclusion criteria in detail.

**Response:** Thank you for pointing this out. To clarify the sample selection process and address your recommendation to describe the inclusion and exclusion criteria, we added the following text to the Methods section (page 4, lines 74–87):

“*To assess sleep-related parameters, we used the Japanese version of the Pittsburgh Sleep Quality Index (PSQI-J). While Cross Marketing Inc. ensured that all questionnaire items were completed (i.e., there were no missing responses), the data from 202 participants were excluded from analysis because of internally inconsistent responses regarding bedtime, wake-up time, and sleep duration, which made it impossible to accurately calculate their global PSQI scores. Responses were considered inconsistent if they included, for example: (1) a reported sleep latency of 120 min, a sleep duration of 8 h, and a total time in bed of only 7 h (i.e., logically impossible); (2) a bedtime of 10:00 PM and a wake-up time of 8:00 AM, but a sleep duration of only 2 h without any reported nighttime awakenings; or (3) a sleep duration that exceeded the total time in bed (e.g., 8.5 h of sleep during 6 h in bed). These types of discrepancies, likely due to input errors or misunderstandings of the questionnaire, prevented the accurate calculation of key PSQI components, such as sleep efficiency. As a result, the final analytical sample consisted of 1,478 participants (728 men, 750 women) who provided logically consistent and complete data on the PSQI-J*.”

2.1) Also, if participants were contacted in a previous data collection, you need to make this explicit in the main text. It should also be made clear whether all of the data collected was collected during the reporting period (from 18 March 2022 to 22 March 2022) or whether some of the information was obtained from the previous data collection.

**Response:** Thank you for your helpful comment. All the data used in our study were collected exclusively for our study during the designated survey period (March 18–22, 2022), and no information from previous surveys was used. To make this clear, we have added the following information to the methods section of the main text (page 4, lines 69–74):

“*The recruitment period for participation in this study was from March 18 to March 22, 2022. Initially, 1,680 participants were selected for the survey. All participants were adults aged 20–70 years, residing in Japan and registered with Cross Marketing Inc. Some participants had previously participated in other surveys; however, the data used in this study were collected specifically for this project during the designated recruitment period, and no data from previous surveys were included.*”

2.2) Has the presence of other medical and/or psychiatric conditions been checked at recruitment? If not, this should be made explicit in the boundaries, as the presence of some of these conditions may affect dream recall.

**Response:** We acknowledge that psychiatric conditions may affect dream recall and appreciate your bringing this to our attention. In response to your suggestion, we added a sentence to the limitations section clarifying that psychiatric conditions were not specifically assessed during participant recruitment, and therefore, their potential influence on the results cannot be excluded. We also emphasized the need to consider these factors in future research. The following sentence was included (page 18, lines 358–361):

“*Moreover, participants in this study were not specifically screened for psychiatric conditions; therefore, the potential influence of such conditions on the results cannot be ruled out. Future studies should consider psychiatric conditions during participant recruitment to better understand their possible impact on dream recall.*”

3) The description of how you scaled the Attitudes toward dreams scale should be included in the main text.

**Response:** Thank you for your helpful comment. In response, we have added a detailed description of the scaling procedure for the Attitudes Toward Dreams Scale in the “Analysis” section (pages 7, lines 156–173). Specifically, we conducted an exploratory factor analysis (EFA) using the principal factor method, removed items with low factor loadings or significant cross-loadings, refined the item pool over five rounds, and performed a confirmatory factor analysis (CFA) to determine the final structure. The final version of the scale comprises two factors with five items each, and each factor’s score was calculated as the mean of the corresponding item scores. We hope this addition clarifies the scaling procedure.

*“The results of the Attitudes Toward Dreams Scale questionnaire were first subjected to exploratory factor analysis (EFA) to extract the factor structure. Items with factor loadings below 0.4 or with significant cross-loadings (i.e., loading differences between factors < 0.1) were systematically removed. After the first round of EFA, four items were excluded. In the second round, three additional items were excluded, followed by one item in the third round and two items in the fourth round. Therefore, a total of 10 items were excluded across four rounds of EFA, resulting in 32 items remaining.*

*After conducting EFA and excluding 10 items, the remaining 32 items were classified into two factors: 22 items in the first factor and 10 items in the second factor. To balance the number of items between the factors, we selected the top 10 items with the highest factor loadings from the first factor. For the second factor, the 10 items retained after EFA were ranked by their factor loadings. Consequently, 10 items were selected from each factor based on the highest loadings, resulting in a 20-item structure. Confirmatory factor analysis (CFA) was then conducted using these 20 items. Subsequently, to identify the model with the best fit, items with the highest factor loadings were selected from both factors, and models with different numbers of items per factor (e.g., eight items, seven items, and five items) were tested. The model with five items per factor demonstrated the highest model fit indices. Therefore, the final version of the Attitudes Toward Dreams Scale consisted of 10 items, with five items representing each factor.”*

Result

4) Age-related ds should also be added to Table 1. Also, Actual% 2020 refers to what? You should describe this in the heading.

**Response:** Thank you for your valuable feedback. To address your comment, we have revised Table 1. We have added the standard deviation for age and clarified the meaning of “Actual% (2020)” in the table’s footnote, indicating that it refers to the age and sex distribution of the Japanese population based on the 2020 national census data (page 8, line 192– page 9, line 195).

5) A table with descriptive analyses of any other measures you collected in the survey should be included.

**Response:** To address this valuable suggestion, we have created a new table (Table 2) that presents the descriptive statistics of the additional measures collected in the survey. Table 2 has been added to the revised manuscript on page 9, line 200 – page 10, line 205.

We have also added a new section titled “Questionnaire scoring and cutoff criteria” to explain the scoring and cutoff values for each item in the survey (pages 5–6, lines 118–133). Furthermore, regarding the STOP-Bang, we have clarified in the “Survey items” section that only four of the eight questions were used for the purpose of our study (page 5, lines 104–107).

“**Questionnaire scoring and cutoff criteria**

*In this study, we used a variety of psychological and sleep-related questionnaires, each with specific scoring characteristics and interpretive guidelines. The Japanese version of the RBD scale (RBDQ-J) serves as a screening tool for REM sleep behavior disorder, with scores of 5 or higher suggesting probable RBD. For the STOP-Bang questionnaire, only the four STOP items (snoring, tiredness, observed apnea, and high blood pressure) were included in this study; therefore, no established cutoff score is applicable. The RLS scale is used to assess symptom frequency, with higher scores indicating more frequent symptoms. The Athens Insomnia Scale is used to screen for insomnia, with scores of 6 or higher suggesting possible insomnia. The PSQI-J is used to measure subjective sleep quality, with a global score of 6 or higher indicating poor sleep quality. The Japanese version of the Metamemory in Adulthood Questionnaire is used to evaluate beliefs and awareness regarding memory functioning, and the NEO-FFI is used to assess five personality traits. In both instruments, higher scores reflect stronger trait expression. The Dream Propensity Scale captures dream-related tendencies; however, no defined cutoff score is provided. The SF-8 is used to measure health-related quality of life using norm-based scoring (mean = 50, standard deviation = 10), with higher scores indicating better perceived health status.*” (pages 5 line 118– page 6 line 133)

“…*questionnaire for the evaluation of obstructive sleep apnea (STOP-Bang) [15] (only the four STOP items: snoring, tiredness, observed apnea, and high blood pressure were included; the BANG items—BMI, age, neck circumference, and gender— were not included and were therefore not obtained from the participants);*” (page 5, lines 104 – 107)

6) Table 4 still shows several measures such as blood pressure, BMI, age, neck circumference, gender, but not the data from these measures?

**Response:** Thank you for highlighting this. We have added a new Table 2 to the manuscript; therefore, the original Table 4 has been renumbered as Table 5 (page 13, line 244 – page 14, line 249).

In this study, we did not administer all items of the STOP-Bang questionnaire; only the four STOP items—snoring, tiredness, observed apnea, and high blood pressure—were included in the survey. Two of the BANG items—BMI and neck circumference—were not part of the questionnaire and were not obtained from the participants. However, age and gender (sex) were collected as part of the basic demographic information and are included in analyses elsewhere in the paper (e.g., comparisons across different age groups and sexes in other tables). The inclusion of these items in the original Table 4 in the context of the STOP-Bang was an error, which we have corrected. We sincerely apologize for this oversight.
